# Calculating the Energy Spectrum of Complex Low-Dimensional Heterostructures in the Electric Field

**DOI:** 10.1155/2013/807462

**Published:** 2013-07-18

**Authors:** Svetlana N. Khonina, Sergey G. Volotovsky, Sergey I. Kharitonov, Nikolay L. Kazanskiy

**Affiliations:** Image Processing Systems Institute of the Russian Academy of Sciences, Molodogvardeiskaya Street 151, Samara 443001, Russia

## Abstract

An algorithm for solving the steady-state Schrödinger equation for a complex piecewise-constant potential in the presence of the *E*-field is developed and implemented. The algorithm is based on the consecutive matching of solutions given by the Airy functions at the band boundaries with the matrix rank increasing by no more than two orders, which enables the characteristic solution to be obtained in the convenient form for search of the roots. The algorithm developed allows valid solutions to be obtained for the electric field magnitudes larger than the ground-state energy level, that is, when the perturbation method is not suitable.

## 1. Introduction

The research aimed at developing high-performance computing systems, communication, and information processing means has led to the emergence of a new approach to designing the electronics components [1–6]. Within such an approach, the information is carried by the amplitude of the electron wave function in a given region of the quantum system. By applying an external *E*-field that introduces changes in the energy spectrum one can induce a controlled redistribution of the system electron density, which corresponds to the data conversion by a predetermined law. 

The devices for controlled electron density redistribution can be physically implemented using structures composed of an array of tunnel-coupled quantum wells. 

In a multiwell quantum structure the wave function amplitude distribution is actually determined by the interference of quantum states of different quantum wells [7]. Because of this, the electron density redistribution under the action of the external *E*-field may appear as a complex, nonmonotonic process. Then, changes in the system's physical characteristics will also be nonmonotonic, thus opening wide opportunities for designing novel quantum devices [8]. 

When designing an electronic device, one needs to learn in which way the energy spectrum of the electric charge can be varied in a desired manner by exposing it to various external actions. The most popular controlling technique is by use of the electric field. In such a system, the energy spectrum can be calculated using the steady-state Schrödinger equation characterized by a designed potential structure and the constant electric field applied. 

For the potential described by a piecewise-constant function, the problem can be solved by representing the wave functions as a superposition of the Airy functions. While simple heterostructures described by one or two potential energy levels enable an analytical solution to be derived, for the complex-shaped potentials the problem becomes very computationally challenging [7]. As a rule, the problem is tackled using the perturbation method [9, 10], which is only suited when the *E*-field applied is low enough. 

Thus, the problem of development and implementation of a numerical method for calculating the energy spectrum of a complex-shaped potential exposed to the electric field remains relevant. 

We describe an algorithm for solving the corresponding steady-state Schrödinger equation. The algorithm is based on consecutively matching the solutions at the zone boundaries with the matrix rank increasing by no more than two orders, thus allowing the procedure for seeking the characteristic equation roots to be easily implemented. 

The comparison of the algorithm with the perturbation method is conducted. 

## 2. An Algorithm for Solving Schrödinger Equation Using the Consecutive Joining 

Let there be a 1D heterostructure composed of an array of homogeneous semiconductor layers (the layer boundaries being perpendicular to the *Ox*-axis) exposed to the *E*-field. If the *E*-field of strength *F* is parallel to the *x*-axis, the potential energy is given by

(1) $$\begin{array}{c} U\left(x\right)=qFx+U_{p}, \end{array}$$

where *q* is the absolute magnitude of the electron charge, *U*_*p*_ is the value of the piecewise-constant potential on the segment  *x* ∈ [*x*_*p*−1_, *x*_*p*_], and *x*_*p*_ are the layer boundary coordinates. 

Then, the Schrödinger equation takes the form

(2) $$\begin{array}{c} -\frac{\hbar^{2}}{2m_{p}}\frac{{\text{d}}^{2}\psi \left(x\right)}{\text{d}x^{2}}+\left(qFx+U_{p}\right)\psi \left(x\right)=E\psi \left(x\right), \end{array}$$

where *m*_*p*_ is the effective mass and *ψ*(*x*) is the particle's wave function.

Denote that

(3) $$\begin{array}{c} u={b_{p}}^{1/3}\left(x-c_{p}\right), \end{array}$$

where *c*_*p*_ = (*E* − *U*_*p*_)/*qF*,  *b*_*p*_ = (2*m*_*p*_ *qF*/*ℏ* ^2^).

In this case, the wave function of the argument *u* satisfies the Airy equation:

(4) $$\begin{array}{c} \left\{\frac{{\text{d}}^{2}}{\text{d}u^{2}}-u\right\}\psi \left(u\right)=0. \end{array}$$

On each interval, the solution of (4) takes the form [11, 12]

(5) $$\begin{array}{c} \psi_{p}\left(u_{p}\left(x\right)\right)=A_{p}Ai\left(u_{p}\left(x\right)\right)+B_{p}Bi\left(u_{p}\left(x\right)\right), \end{array}$$

where *u*_*p*_(*x*) = *b*_*p*_^1/3^(*x* − *c*_*p*_), *x* ∈ [*x*_*p*−1_, *x*_*p*_];  *Ai*(*x*), *Bi*(*x*) are the Airy functions of the first and second kind, respectively. 

By imposing the matching conditions of the wave functions and derivatives thereof divided by the mass on the interval (layer) boundaries, the coefficients of the solution in (5) can be represented as

(6) ApAi(up,p)+BpBi(up,p)  =Ap+1Ai(up+1,p)+Bp+1Bi(up+1,p),1mp[ApAi′(up,p)+BpBi′(up,p)]  =1mp+1[Ap+1Ai′(up+1,p)+Bp+1Bi′(up+1,p)],

where *u*_*p*,*l*_ = *b*_*p*_^1/3^(*x*_*l*_ − *c*_*p*_).

Note that the allowed values of energy *E*  implicitly enter in (6) in the form of the Airy function arguments in (5). In the following, we consider obtaining the characteristic equation (for *E*) by a simple example of an infinite quantum well and a general-form quantum well potential. 

## 3. Infinite Triangular Potential Well

**Figure 1:**
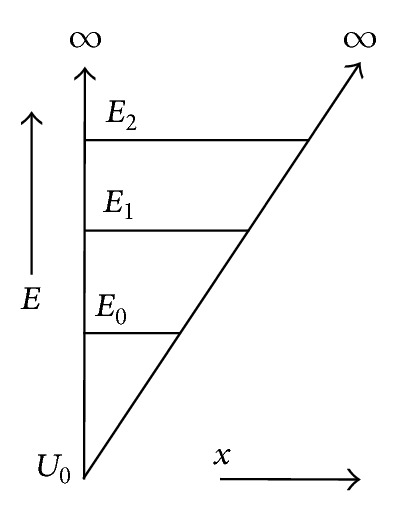
The energy spectrum of an infinite triangular potential well.

By way of illustration, the model of a triangular infinite potential well is utilized when describing the surface quantization. The potential of the infinite triangular well is described by the relation (Figure 1)

(7) $$\begin{array}{c} U\left(x\right)=\{\begin{array}{cc} \infty , & x\in \left[-\infty ,x_{0}\right],\\ U_{0}+qFx, & x\in \left[x_{0},\infty \right]. \end{array} \end{array}$$

The boundary conditions define that the wave function in (5) has the zero value at the left boundary of the well (when *x* = *x*_0_ and *x* → *∞*). Whence it follows that  *B*_0_ = 0, and thus,

(8) $$\begin{array}{c} \psi_{0}\left(u\left(x_{0}\right)\right)=A_{0}Ai\left(u\left(x_{0}\right)\right)=0, \end{array}$$

where *u*(*x*_0_) = (*x*_0_ − (*E* − *U*_0_)/*qF*)(2*m*_0_ *qF*/*ℏ* ^2^)^1/3^.

The constant *A*_0_ in (8) is normalized on the assumption that the integral of the wave function's squared modulus is equal to unity. 

The condition in (8) holds when *u*(*x*_0_) = *a*_*n*_, where *a*_*n*_ are the Airy function roots. Thus, the allowed energy values are

(9) $$\begin{array}{c} E_{n}=\left[x_{0}-a_{n}\cdot {\left(\frac{2m_{0}qF}{\hbar^{2}}\right)}^{-1/3}\right]qF+U_{0}. \end{array}$$

Putting *x*_0_ = 0 and using the approximate values of the Airy function roots [11, 12]

(10) $$\begin{array}{c} a_{n}\approx -{\left[\left(\frac{3\pi }{8}\right)\left(4n-1\right)\right]}^{2/3}, \end{array}$$

we obtain an approximate estimate of the energy spectrum of the infinite triangular well in the explicit form (with regard to the electron charge)

(11) $$\begin{array}{c} E_{n}\approx {\left(\frac{\hbar^{2}}{2m_{0}}\right)}^{1/3}{\left[\frac{3}{2}qF\pi \left(n-0,25\right)\right]}^{2/3}+U_{0}. \end{array}$$

In the following, we conduct the comparison of the energy spectra of a finite-width square well with and without the *E*-field applied. 

## 4. Infinite Square Potential Well

**Figure 2:**
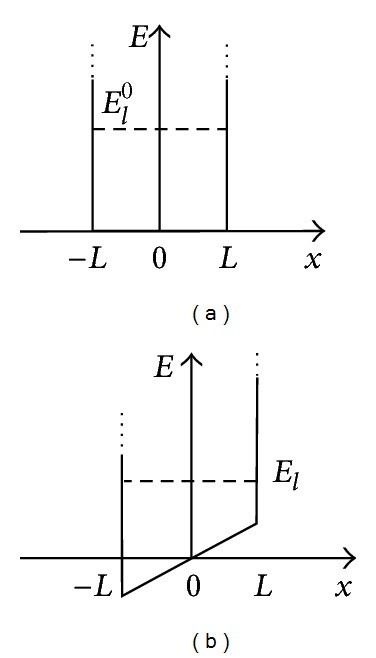
The infinite square potential well (a) with and (b) without the *E*-field applied.

For a quantum well of width 2*L*, the potential is (Figure 2)

(12) $$\begin{array}{c} U\left(x\right)=\{\begin{array}{cc} \infty , & x\in \left[-\infty ,-L\right],\\ U_{0}+qFx, & x\in \left[-L,L\right],\\ \infty , & x\in \left[L,\infty \right]. \end{array} \end{array}$$

For a classical infinite square quantum well of width 2*L*, the solution is known to take the form

(13) $$\begin{array}{c} \psi_{n}\left(x\right)=\sin\left(\alpha_{n}\left(x+L\right)\right),\;\; \end{array}$$

where

(14) $$\begin{array}{c} \alpha_{n}=\sqrt{\frac{2m_{0}}{\hbar^{2}}\left(E_{n}-U_{0}\right)},\;\;E_{n}={\left(\frac{\pi n}{2L}\right)}^{2}\frac{\hbar^{2}}{2m_{0}}+U_{0}. \end{array}$$

When applying a low-value *E*-field, an approximate solution can be derived using the perturbation method [9, 10].

### 4.1. Solving the Schrödinger Equation by the Perturbation Method

Let us consider the perturbation theory for a nondegenerate state. The steady-state Schrödinger equation (2) can be written as

(15) $$\begin{array}{c} H\left[\varphi_{n}\left(x\right)\right]=E_{n}\varphi_{n}\left(x\right), \end{array}$$

where the operator *H*  takes the form

(16) $$\begin{array}{c} H=H_{0}+W, \end{array}$$

*W* is the perturbation operator and  *H*_0_  is the nonperturbed operator whose eigenfunctions and eigenvalues are defined by (13) and (14):

(17) $$\begin{array}{c} H_{0}\left[\psi_{n}\left(x\right)\right]=E_{n}^{0}\psi_{n}\left(x\right). \end{array}$$

The sought-for eigenfunction of the perturbed operator can be decomposed in terms of the unperturbed operator as

(18) $$\begin{array}{c} \varphi_{n}\left(x\right)=\underset{m}{\sum }c_{n}^{m}\psi_{m}\left(x\right). \end{array}$$

Substituting (18) into (15) yields

(19) $$\begin{array}{c} \underset{m}{\sum }c_{n}^{m}W\left[\psi_{m}\left(x\right)\right]=\underset{m}{\sum }c_{n}^{m}\left(E_{n}-E_{m}^{0}\right)\psi_{m}\left(x\right). \end{array}$$

Taking the scalar product of (19) by *ψ*_l_*(*x*)  and with regard to the orthogonality, we obtain

(20) ∑mcnm∫ψl∗(x)W[ψm(x)]dx=cnl(En−El0),                  l=1,2,3,….

Assuming that the perturbation operator is infinitesimal, we find the energy levels and wave functions of the perturbed and unperturbed operators to be close to each other. The sought-for solution will include the second-order corrections:

(21) $$\begin{array}{c} E_{n}\approx E_{n}^{0}+E_{n}^{1}+E_{n}^{2},\\ \varphi_{n}\left(x\right)\approx \psi_{n}\left(x\right)+\underset{m\neq n}{\sum }c_{n}^{m,1}\psi_{m}\left(x\right)+\underset{m\neq n}{\sum }c_{n}^{m,2}\psi_{m}\left(x\right). \end{array}$$

From (20) and (21), the corrections are described by the following equations [9, 10]:

(22) En1=∫ψn∗(x)W[ψn(x)]dx,En2=∑m≠ncnm,1∫ψn∗(x)W[ψm(x)]dx,cnm,1=∫ψm∗(x)W[ψn(x)]dx(En0−Em0),cnm,2={∑lcnl,1∫ψl∗(x)W[ψl(x)]dx−cnm,1En1} ×(En0−Em0)−1.

### 4.2. Perturbations Method for an Infinite Square Potential Well in the Electric Field

In the case in question,

(23) $$\begin{array}{c} H_{0}=-\frac{\hbar^{2}}{2m_{0}}\frac{{\text{d}}^{2}}{\text{d}x^{2}}+U_{0},\\ W=qFx. \end{array}$$

The unperturbed solutions are considered to be given by (13) and (14). In this case, the energy values for (2) are derived from the relation:

(24) $$\begin{array}{c} E_{n}\approx {\left(\frac{\pi n}{2L}\right)}^{2}\frac{\hbar^{2}}{2m_{0}}+U_{0}+E_{n}^{1}+E_{n}^{2},\;\; \end{array}$$

where

(25) En1=qF∫−LLsin2(αn(x+L))x dx=qFL,En2=qF∑mcnm,1∫−LLsin(αn(x+L))sin(αm(x+L))x dx −cnn,1En1.

Then, the wave functions are determined as follows:

(26) φn(x)=sin[(x+L)2m0ℏ2(En−U0)] +∑m≠ncnm,1ψm(x)+∑m≠ncnm,2ψm(x),

where

(27) cnm,1=qF∫−LLsin(αm(x+L))sin(αn(x+L))x dx(En0−Em0)  ,cnm,2={qF∑lcnl,1∫−LLsin(αm(x+L))×sin(αl(x+L))x dx−cnm,1En1}×(En0−Em0)−1.

For the first-order approximation, all allowed energy levels in the infinite well are shifted by the same value  *qF* *L*, whereas for the second-order approximation the gap between the quantum well bottom and the ground state will decrease as the square of the *E*-field strength.

The perturbation method remains suitable until the maximal change of potential at the well boundary due to the *E*-field reaches the order of the ground state energy. If the *E*-field applied becomes larger, the direct matching algorithm described in Section 1 needs to be used. 

### 4.3. The Matching Method for an Infinite Potential Well in the *E*-Field

The boundary conditions are derived on the assumption that the wave function of (5) has a zero value at the well boundaries:

(28) $$\begin{array}{c} \psi_{0}\left(u_{-L}\right)=A_{0}Ai\left(u_{-L}\right)+B_{0}Bi\left(u_{-L}\right)=0,\\ \psi_{0}\left(u_{L}\right)=A_{0}Ai\left(u_{L}\right)+B_{0}Bi\left(u_{L}\right)=0, \end{array}$$

where

(29) $$\begin{array}{c} u_{-L}=\left(-L-\frac{E-U_{0}}{qF}\right){\left(\frac{2m_{0}qF}{\hbar^{2}}\right)}^{1/3},\\ u_{L}=\left(L-\frac{E-U_{0}}{qF}\right){\left(\frac{2m_{0}qF}{\hbar^{2}}\right)}^{1/3}. \end{array}$$

Thus, we obtain a homogeneous equation

(30) $$\begin{array}{c} \left(\begin{array}{cc} Ai\left(u_{-L}\right) & Bi\left(u_{-L}\right)\\ Ai\left(u_{L}\right) & Bi\left(u_{L}\right) \end{array}\right)\left(\begin{array}{c} A_{0}\\ B_{0} \end{array}\right)=0, \end{array}$$

which has a nontrivial solution if the determinant is equal to zero:

(31) $$\begin{array}{c} Ai\left(u_{-L}\right)Bi\left(u_{L}\right)-Ai\left(u_{L}\right)Bi\left(u_{-L}\right)=0. \end{array}$$

This equation determines the eigenvalues  *E*_*n*_.

The coefficient  *B*_0_ can be expressed through *A*_0_ using one of the equations in (28):

(32) $$\begin{array}{c} B_{0}=-A_{0}\frac{Ai\left(u_{-L}\right)}{Bi\left(u_{-L}\right)}=-A_{0}\frac{Ai\left(u_{L}\right)}{Bi\left(u_{L}\right)}, \end{array}$$

with the value of the coefficient  *A*_0_ derived from the wave-function normalization condition. 

In the following, we conduct the comparison of the solutions derived by the two methods. 

### 4.4. Comparison of the Results Derived by the Two Methods

The parameters used in the calculations are as follows:  *m*_0_ = 0,1*m*_*e*_, *m*_*e*_ = 9.10938188 × 10^−31^ kg is the electron mass, *ℏ* = 1.054571726(47) × 10^−34^ J·s is the Planck constant, and the potential *U* and energy *E* are in electron-volts (1 eV = 1.602176487(40) × 10^−19^ J), with the *E*-field given in the reduced values. In the case of interest, *U*_0_ = 0 and the well width is  2*L* = 2 nm. 

Table 1 gives the values of the first three allowed energy states for an infinite square well in the absent *E*-field, in the weak *E*-field and in the “strong” *E*-field (i.e., when the *E*-field strength is higher than the ground state energy).

Table 1 suggests that when applied for a strong *E*-field, the perturbations method produces invalid results. The matching method leads to widened band gaps between the energy states as the *E*-field applied is increasing. Note that applying the strong *E*-field results in the narrowed gap between the well bottom and the ground state energy. 

**Figure 3:**
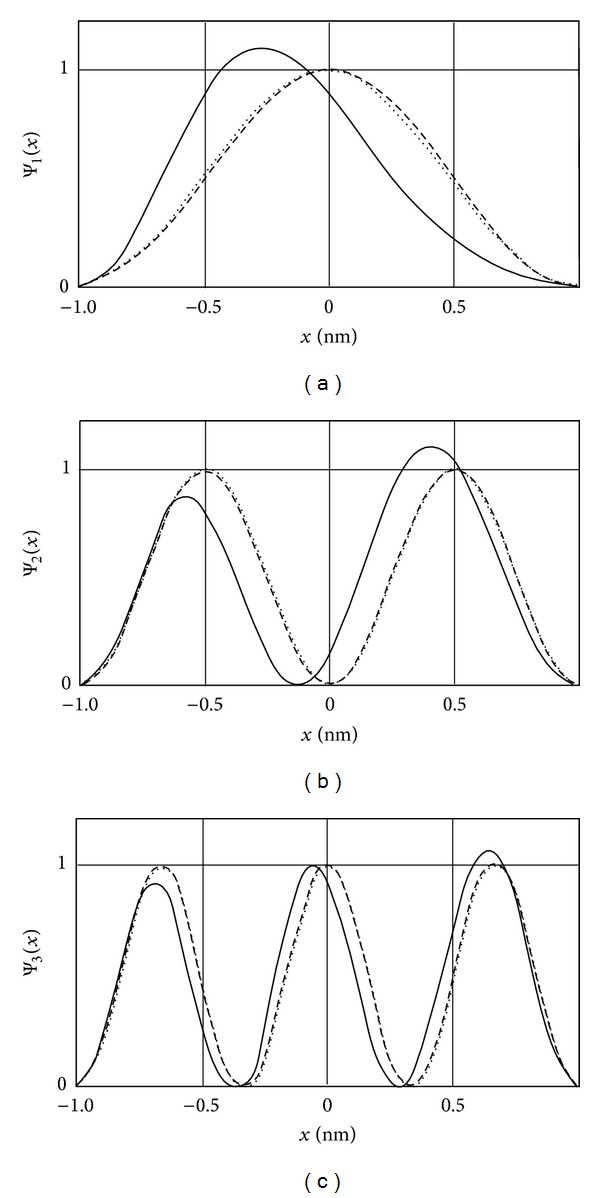
Wave functions for the infinite well (zero field—dashed line,  *qF* = 0.1—dotted line, and  *qF* = 2—solid line) at *n* = 1 (a), *n* = 2 (b), and *n* = 3 (c).

Figure 3 shows the first three wave functions in the absence of the *E*-field, in the weak *E*-field and in the strong *E*-field derived using the matching method. According to Figure 3, with increasing *E*-field, the probability of the electron to be found in the potential well ceases to be symmetric, being shifted toward one of the well boundaries. 

## 5. Infinite Square Well with a General-Form Piecewise-Linear Potential 

In the general case, the potential of an infinite well in the *E*-field is described as

(33) $$\begin{array}{c} U\left(x\right)=\{\begin{array}{cc} \infty , & x\in \left[-\infty ,-L\right],\\ U_{0}+qFx, & x\in \left[-L,x_{0}\right],\\ \vdots & \\ U_{p}+qFx, & x\in \left[x_{p-1},x_{p}\right],\\ \vdots & \\ U_{N}+qFx, & x\in \left[x_{N-1},L\right],\\ \infty , & x\in \left[L,\infty \right]. \end{array} \end{array}$$

For the external bands, the conditions of the zero-value wave function in (5) at the well boundaries need to be met:

(34) $$\begin{array}{c} A_{0}Ai\left(u_{-L}\right)+B_{0}Bi\left(u_{-L}\right)=0,\\ A_{N}Ai\left(u_{L}\right)+B_{N}Bi\left(u_{L}\right)=0, \end{array}$$

whereas for the internal bands, the matching conditions of (6) should be valid:

(35) [Ai(up,p)Bi(up,p)Ai′(up,p)mpBi′(up,p)mp](ApBp)  =[Ai(up+1,p)Bi(up+1,p)Ai′(up+1,p)mp+1Bi′(up+1,p)mp+1](Ap+1Bp+1),

where the arguments *u*_*p*,*l*_, *u*_−*L*_, *u*_*L*_ are defined by (3).

The last-band (-layer) coefficients can be expressed through  *A*_0_ and *B*_0_ as

(36) $$\begin{array}{c} \left(\begin{array}{c} A_{N}\\ B_{N} \end{array}\right)=Q_{N-1}Q_{N-2}\cdot \ldots \cdot Q_{1}\left(\begin{array}{c} A_{0}\\ B_{0} \end{array}\right),\;\; \end{array}$$

where

(37) Qp=[Ai(up+1,p)Bi(up+1,p)Ai′(up+1,p)mp+1Bi′(up+1,p)mp+1]−1 ×[Ai(up,p)Bi(up,p)Ai′(up,p)mpBi′(up,p)mp].

Thus, for the coefficients *A*_0_, *B*_0_, *A*_*N*_, and *B*_*N*_ we have derived four equations in (34), (36), which form a homogeneous linear system. Putting the system's determinant equal to zero, we obtain a characteristic equation for deriving the eigenvalues of *E*.

Applying the algorithm of (34)–(37) for the consecutive matching of solutions at the band boundaries with use of the second-rank matrix, the characteristic equation can be put in a more convenient form. 

The numerical implementation of the algorithm (34)–(37) allows a simple solution of the steady-state Schrödinger equation (2) with the complex potential of (33) to be derived. However, this calls for the use of the “exponential arithmetic” (arithmetic over numbers represented as *a* exp(*b*), where *a* and *b* are the number parameters). Otherwise, the software implementation will be incorrect at small values of the *E*-field: *qF* < 0.5.

**Figure 4:**
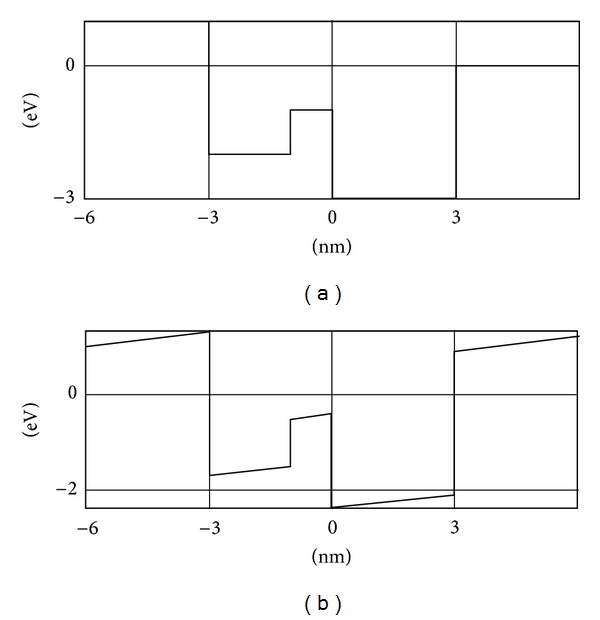
Complex-form potential without (a) and with the *E*-field applied: *qF* = 0.1 (b).

**Figure 5:**
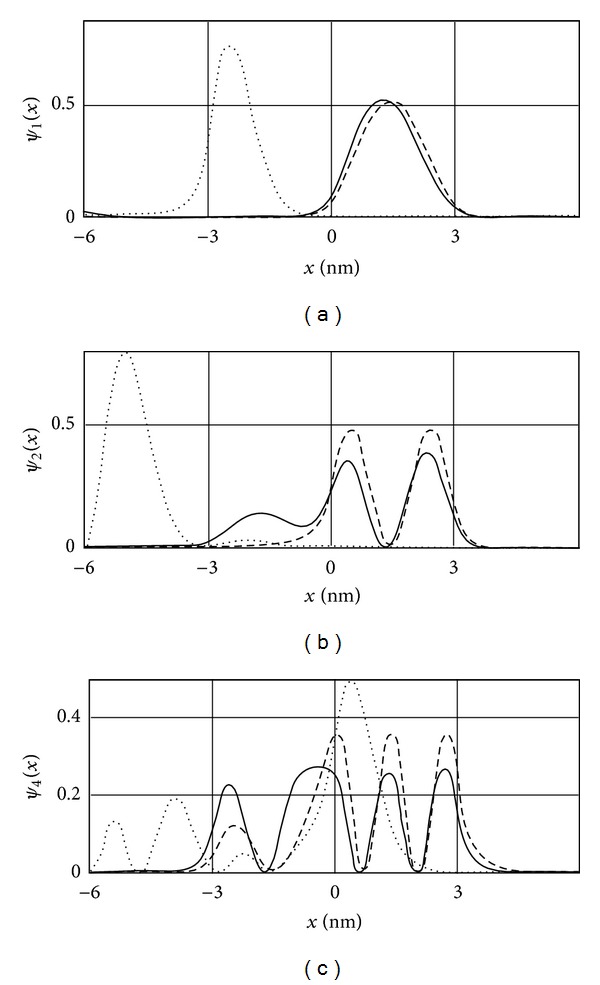
Wave functions for a complex relief (zero *E*-field—dashed line, and  *qF* = 0,1—solid line,  *qF* = 1—dotted line) for *n* = 1 (a), *n* = 2 (b), and *n* = 4 (c).

Figure 4 depicts a complex-form potential with and without the *E*-field applied.  Table 2 gives the corresponding values of the energy spectrum for the said potential. The wave functions distributions are shown in Figure 5. 

The computation results suggest that by varying the potential form and the external *E*-field both the energy spectrum and the distribution of the probability of finding the particle in a definite heterostructure region can be essentially varied. 

## 6. Conclusions

We have developed and implemented an algorithm for solving the steady-state Schrödinger equation for a complex piecewise-constant potential in the presence of the *E*-field. The algorithm is based on the consecutive matching of solutions given by the Airy functions at the band boundaries with the matrix rank not exceeding two, thus allowing the characteristic equation to be derived in the convenient form for the search of the roots. 

It has been numerically shown that the algorithm developed allows valid solutions to be derived when the value of the *E*-field applied is larger than the ground-state energy level, that is, under the conditions when the perturbation method is inapplicable. 

The computation results obtained for the complex potential distribution have shown that by varying the potential profile and the value of the *E*-field applied it becomes possible to essentially vary the energy spectrum and the probability of finding the particle in one or another region of the heterostructure. 

## Figures and Tables

**Table 1:** Energy spectrum (first three values) for a square well.

	Zero *E*-field	Field *qF* = 0.1		Field *qF* = 2	
		Perturbations method	Matching method	Perturbations method	Matching method
*E*_1_	0.938357	1.037995	0.937895	2.755581	0.761992
*E*_2_	3.753428	3.853667	3.753566	5.810891	3.809982
*E*_3_	8.445214	8.545637	8.445296	10.576528	8.494495

**Table 2:** Energy spectrum (first 9 values) for a complex relief.

	Zero	*E*-field	*E*-field
	*E*-field	*qF* = 0.1	*qF* = 1
*E*_*n*_	−2.741001	−2.602847	−3.705108
	−2.000144	−1.883931	−3.295242
	−1.577124	−1.733931	−2.376165
	−0.900418	−0.851777	−1.857336
	−0.355715	−0.422565	−1.647189
	0.305344	0.493440	−0.779213
	0.660222	0.833417	−0.338370
	1.210436	1.425745	0.357965
	1.492179	1.979392	0.942955
	*⋯*	*⋯*	*⋯*
